# Carbamoylase-based impedimetric electronic tongue for rapid detection of paralytic shellfish toxins

**DOI:** 10.1007/s00216-024-05199-8

**Published:** 2024-02-15

**Authors:** Mariana Raposo, Silvia Soreto, Catarina Moreirinha, Maria Teresa S. R. Gomes, Sara T. Costa, Maria João Botelho, Bruno M. G. Melo, Luís Cadillon Costa, Alisa Rudnitskaya

**Affiliations:** 1 CESAM and Chemistry Department, University of Aveiro, 3810-193 Aveiro, Portugal; 2 I3N and Department of Physics, University of Aveiro, 3810-193 Aveiro, Portugal; 3 IPMA, Portuguese Institute for the Sea and Atmosphere, 1449-006 Lisbon, Portugal; 4 CIIMAR, Interdisciplinary Centre of Marine and Environmental Research, University of Porto, 4450-208 Matosinhos, Portugal; 5 ICBAS, School of Medicine and Biomedical Sciences, University of Porto, 4050-313 Porto, Portugal

**Keywords:** Carbamoylase, Paralytic shellfish toxins, Electrochemical impedance spectroscopy, Electronic tongue

## Abstract

Phytotoxins produced by marine microalgae, such as paralytic shellfish toxins (PSTs), can accumulate in bivalve molluscs, representing a human health concern due to the life-threatening symptoms they cause. To avoid the commercialization of contaminated bivalves, monitoring programs were established in the EU. The purpose of this work is the implementation of a PST transforming enzyme—carbamoylase—in an impedimetric test for rapid simultaneous detection of several carbamate and N-sulfocarbamoyl PSTs. Carbamoylase hydrolyses carbamate and sulfocarbamoyl toxins, which may account for up to 90% of bivalve toxicity related to PSTs. Conformational changes of carbamoylase accompanying enzymatic reactions were probed by Fourier transform mid-infrared spectroscopy (FT-MIR) and electrochemical impedance spectroscopy (EIS). Furthermore, a combination of EIS with a metal electrode and a carbamoylase-based assay was employed to harness changes in the enzyme conformation and adsorption on the electrode surface during the enzymatic reaction as an analytical signal. After optimization of the working conditions, the developed impedimetric e-tongue could quantify N-sulfocarbamoyl toxins with a detection limit of 0.1 µM. The developed e-tongue allows the detection of these toxins at concentration levels observed in bivalves with PST toxicity close to the regulatory limit. The quantification of a sum of N-sulfocarbamoyl PSTs in naturally contaminated mussel extracts using the developed impedimetric e-tongue has been demonstrated.

**Supplementary Information:**

The online version contains supplementary material available at 10.1007/s00216-024-05199-8.

## Introduction

The out-of-control proliferation of (micro)algae or cyanobacteria with concomitant toxic and harmful effects, also called harmful algal blooms (HABs), is a phenomenon that occurs in most coastal countries. During HABs, some of these microalgae species biosynthesize toxins that can affect marine organisms and can also be accumulated in the tissues of filter-feeding bivalves [1, 2]. The subsequent consumption of contaminated seafood poses a serious threat to human health as it may provoke shellfish poisoning. The impacts of toxic algal blooms also include economic losses in industries such as aquaculture and tourism due to harvesting closures. As occurrences of HABs are unpredictable, routine monitoring programs of toxins for commercial bivalve species were established in the EU countries, including Portugal [3, 4].

Main marine toxins included in the monitoring programs worldwide are categorized according to the symptoms they cause in humans: paralytic shellfish toxins (PSTs), amnesic shellfish toxins (ASTs) and diarrhetic shellfish toxins (DSTs) besides some other lipophilic toxins [4]. Toxic episodes caused by PSTs are less frequent in Portuguese coastal waters; however, among the monitored marine toxins, they are of particular concern due to their harmful effects on humans. Those effects include a range of neurological symptoms, in severe cases leading to respiratory failure [5]. PSTs are a large group of compounds, all having the same mode of action (inhibition of the voltage-gated sodium channel in excitable cells) but with different structures and toxicities [5]. Currently, more than fifty toxins have been reported that are divided into sub-groups depending on the substituent side chains they have, such as carbamate, sulphate, hydroxyl, hydroxybenzoate or acetate [6].

According to European Union regulations, the current officially accepted method for PST quantification in bivalves is the liquid chromatography (LC) with fluorometric detection (FLD) [7]. However, LC-FLD is a technique that requires costly equipment and specialized personnel to operate, and it needs laborious and time-consuming sample preparation. Significant efforts have been directed to the development of less expensive and simpler methods for PST detection and quantification, including several biosensors and immunoassays along with nerve cell and sodium channel-based assays [8, 9]. Antibodies employed in immunoassays and biosensors are mostly produced against saxitoxin (carbamoyl group), and as a result, they display low affinity to PSTs from other groups, such as N-sulfocarbamoyl [10, 11], a toxin group that dominates toxin profiles produced by the dinoflagellate *Gymnodinium catenatum* [12]. Though *G. catenatum* is less common compared to the other PST-producing dinoflagellates, it has a worldwide distribution ranging from the Gulf of California to the Pacific coast of South America and from Asia to the Mediterranean and the Atlantic coasts of Spain, Portugal and Morocco [13]. Alternative methods of PST quantification in bivalves, such as the receptor-based and nerve cell methods, have the advantage of directly producing toxicity estimates of PSTs, which is well correlated with animal tests. However, they involve laborious preparation procedures, have long response times and, most importantly, suffer from low stability and reproducibility [14–16]. Thus, the development of alternative sensing methodologies not involving the use of antibodies or cells for PSTs’ detection is of practical interest.

In our previous work, a range of potentiometric chemical sensors with plasticized PVC membranes displaying sensitivity to four PSTs commonly found in Portuguese waters, namely, gonyautoxin 5 (GTX5), N-sulfocarbamoyl gonyautoxins 2&3 (C1&2), decarbamoyl saxitoxin (dcSTX) and saxitoxin (STX), has been developed [17]. Low selectivity of the sensors to the studied toxins was observed; for example, most sensors were not selective either to saxitoxin or decarbamoyl saxitoxin with selectivity coefficients being close to 0, while two sensors displayed higher selectivity towards decarbamoyl saxitoxin, being capable of detecting it in the presence of STX in 25 and 4 times excess [17]. Low selectivity of the sensors makes difficult to use them for selective simultaneous quantification of multiple PSTs. Nonetheless, this low selectivity accompanied by a high sensitivity makes these sensors suitable candidates for the development of an electronic tongue sensor system. E-tongues are multisensor systems that by combining an array of partially selective sensors and chemometric tools for data processing enable a quantitative analysis and an accurate classification of multicomponent media [18]. Thus, an e-tongue based on 6 potentiometric sensors was developed and applied to the simultaneous quantification of three toxins, dcSTX, GTX5 and C1&2, in bivalve extracts [19]. However, low selectivity of the sensors to N-sulfocarbamoyl toxins GTX5 and C1&2 in the presence of dcSTX remained an issue leading to higher errors of these PSTs’ quantification. To improve quantification precision of N-sulfocarbamoyl toxins, an enzymatic assay has been proposed [20]. Enzymatic assay employed an enzyme — carbamoylase — that hydrolyses the carbamoyl and N-sulfocarbamoyl groups of PSTs transforming carbamate and N-sulfocarbamoyl toxins in their respective decarbamoylated analogues [6]. Carbamoylase is produced by several clam species including the surf clam *Spisula solida* [21, 22] that inhabits the Portuguese coast [20] and was used as a source of enzyme in this work. The enzymatic assay involved the hydrolysis of GTX5 by carbamoylase followed by the quantification of the product of enzymatic reaction, dcSTX, using a potentiometric chemical sensor with selectivity to dcSTX in the presence of GTX5.

Electrochemical impedance spectroscopy (EIS) is a powerful technique for probing interactions on the sample-electrode interface and for the transduction of biosensing events at electrodes. EIS has found numerous applications in biosensing in combination with enzymes, antibodies, DNA and cells [23, 24]. The first impedimetric e-tongue comprised six interdigitated electrodes modified with conducting polymers deposited by Langmuir–Blodgett or layer-by-layer techniques [25]. Further on, several impedimetric e-tongues based on arrays of metal electrodes [26], electrodes modified with nanomaterials [27] and fragments of antimicrobial proteins [28] were reported with applications ranging from foodstuff analysis to microbial endotoxins detection. Recently, the use of the whole impedance spectra for data processing gave rise to the concept of one sensor impedimetric tongue setup [29]. In this work, the impedimetric spectra of different coffee brews were measured using label-free carbon paste electrode. Classification models for the detection of coffee adulteration were calculated using complex number-supervised pattern recognition methods such as partial least-squares discriminant analysis (PLS-DA) and soft independent modelling by class analogy (SIMCA) [29].

The purpose of the present study was to continue the work on the development of tools for rapid toxin detection focusing on detection of a sum of carbamate and N-sulfocarbamoyl toxins, which may account for up to 90% of bivalve toxicity related to PSTs [30]. In this work, a PST transforming enzyme — carbamoylase — extracted from *S. solida* was implemented in the assay with one sensor for impedimetric detection of a sum of N-sulfocarbamoyl and carbamate PSTs.

## Material and methods

### Reagents and materials

Sodium hydrogen phosphate, ammonium formate, acetic acid and dihydrogen phosphate were from Sigma Aldrich Química, S.L. (Algés, Portugal); acetonitrile (LC grade) was from Riedel-de Haën (Germany); sodium chloride and sodium hydroxide were from Merck Life Science S.L.U. (Portugal); hydrogen peroxide was from Carlo Erba (France); methanol was from Fisher (Trinidad and Tobago); ethylenediaminetetraacetic acid dipotassium salt was from Panreac Química S.L.U. (Spain); ammonium sulphate and aprotinin were from VWR International – Material de Laboratório, Lda (Alfragide, Portugal); and bestatin was from Santa Cruz Biotechnology Inc. (Heidelberg, Germany). Pierce™ BCA Protein Assay Kit was from Thermo Fisher Scientific (Porto Salvo, Portugal). Octadecyl bonded phase silica C18 solid-phase extraction cartridges (500 mg/3 mL) were from Supelclean (Supelco, USA); HiPrep Octyl FF (High Sub) 16/10 column was from Cytiva (VWR – International Lda., Carnaxide, Portugal); Amicon Ultra centrifugal filter units, ultra-15, MWCO 30 kDa were from Millipore (Merck KGaA, Darmstadt, Germany). All reagents were p.a. (for analysis) grade unless stated otherwise.

Screen-printed electrodes (SPE) with gold, carbon or carbon modified with polyaniline (PANI) working electrodes, gold or carbon auxiliary electrodes and silver reference electrodes were from DropSens (Spain). SPE were fabricated on ceramic substrate with sizes: L33 × W10 × H0.5 mm with circular working electrode with diameter 4 mm.

Standard solutions of PSTs (dcSTX, GTX5 and C1&2) were certified reference material from the Cifga Laboratories (Lugo, Spain). When working with PSTs, a long-sleeved lab coat and non-permeable nitrile or latex gloves were used. Toxin containing waste was decontaminated using a 10% solution of sodium hypochlorite for 30 min and disposed of down the drain with plenty of water.

Ultrapure water produced by Merck Millipore Water System (18 MΩcm^−1^) was used for all solution preparation.

### Carbamoylase extraction and quantification

Enzyme carbamoylase was extracted from the crystalline style tissues dissected from surf clam (*S. solida*) specimens obtained from a local fisherman. Extraction and purification procedures were adopted from [20, 31]. Briefly, ca. 10 g of crystalline style was homogenized at 4°C with approximately 10 volumes of 50 mmol L^−1^ phosphate buffer (pH 7.0) containing 0.5 mol L^−1^ of ammonium sulphate, 5 mg L^−1^ of aprotinin, 5 mg L^−1^ of bestatin and 0.1 mmol L^−1^ of EDTA. After a first centrifugation at 4 °C and 550 × g (30 min) followed by 7500 × g (30 min) and filtration of the supernatant through 10-µm membrane, the crude crystalline style extract was obtained. The extract was loaded onto an octyl sepharose 4 fast-flow column previously equilibrated with 50 mmol L^−1^ phosphate buffer (pH 7.0) containing 0.7 mol L^−1^ of NaCl. The column was eluted stepwise with five column volumes of 50 mmol L^−1^ phosphate buffer (pH 7.0) and ending with deionized water at a flow rate of 2.5 mL min^−1^. The 50 mmol L^−1^ phosphate buffer eluate was concentrated by ultrafiltration using a 30 KDa filter. Enzyme extracts in the 50 mmol L^−1^ phosphate buffer were stored at − 80 °C prior to use.

Enzyme concentration in extracts was determined using a protein quantification kit, based on the Smith method according to the instructions of the manufacturer [32]. Enzymatic assays for the evaluation of the activity of the carbamoylase towards GTX5 and C1&2 were done by mixing toxin certified reference solutions with 100 µL of enzyme extract to obtain 1 µmol L^−1^ toxin concentration. Incubations were done in the same conditions used in our previous study [20], namely, reaction time 3 h and temperature 25 °C. Enzymatic reactions were stopped by adding 10 µL of 10 mol L^−^.^1^ acetic acid and freezing at − 18 °C until toxin analysis. Assays were run in triplicate. Substrate consumption was found to be around 70%, which is in agreement with the previously reported results [20].

### Bivalve extract preparation

Extracts of mussel whole soft tissues were prepared: two extracts corresponded to bivalve specimens that have been naturally contaminated with PSTs during *G. catenatum* blooms and another one to organisms not exposed to PSTs. The contaminated specimens were harvested during PST outbreaks at Sagres coastal area and Aveiro lagoon, Portugal, in 2012 and 2015, respectively. Mussels free of PSTs were harvested in 2016 at Aveiro lagoon in the period of the absence of blooms. Specimens were sacrificed and dissected, and three composite samples (*n* = 20) of whole soft tissues (contaminated and free of PSTs) were prepared and stored at − 25 °C until further analysis.

Extract preparation was carried out according to the official AOAC method for PST quantification [33], which consists in two steps, an acid extraction and a clean-up. A detailed description of procedures can be found elsewhere [13]. The pH of the extracts was adjusted to 6.5 with 0.2 mol L^−1^ NaOH before oxidation reaction and subsequent measurements.

### Quantification of PSTs by LC-FLD

Quantification of PSTs in bivalve extracts and in assays for evaluation of the activity of the carbamoylase was based on the official AOAC method [33]. A procedural modification in the oxidation of PSTs was introduced due to the dominance of N-sulfocarbamoyl and decarbamoyl compounds in the *G. catenatum* toxic profile [34]. Sample oxidation procedure for the bivalve extracts, chromatographic conditions and details in PST quantification are described in Costa ST et al. [35].

Total toxicity values in bivalve extracts were estimated in terms of µg STX di-HCl equivalents per kg of bivalve tissue, multiplying the toxin concentration by the toxicity equivalence factor (TEF) of each individual compound [36]. In the case of isomeric pairs such as dcGTX2&3 and C1&2, the highest TEF was used for each pair. The regulatory limit (RL) for PSTs is 800 µg STX di-HCl equivalents per kg of bivalve tissue [36].

### Electrochemical impedance spectroscopy measurements

Complex impedance (Z^*^) measurements for frequencies from 100 Hz up to 1 MHz were carried out using a Solartron Analytic Modulab XM MTS with a signal amplitude of 100 mV, at room temperature (25 °C). A drop of 100 µL of a solution was placed on the surface of the SPE, and impedance spectra were recorded. The electrode surface was washed with ultrapure water between measurements.

Firstly, measurements were carried out using SPE with different working electrodes (gold, carbon and carbon modified with PANI). Measurements were made in the phosphate buffer and solutions of enzyme (2.7 mg mL^−1^), toxins dcSTX and GTX5 (both at 3 µmol L^−1^) and a mixture of solutions of enzyme and GTX5. Solutions were prepared in the 50 mmol L^−1^ phosphate buffer with pH 7.0 containing 1 mmol L^−1^ of NaCl. Incubation time for the enzymatic assay was 30 min.

Further experiments were carried out using SPE with gold working electrode. The optimization of the enzymatic assay conditions was carried out by varying concentrations of enzyme and GTX5 and incubation time according to the fractional factorial experimental design (Table 1). Further optimization of the enzyme concentration was done by measuring a series of assays with GTX5 concentration of 0.3 µmol L^−1^, reaction time of 5 min and enzyme concentration varying from 0.05 to 2 mg mL^−1^.

**Table 1:** Experimental plan for the optimization of the impedimetric e-tongue working conditions

Run	GTX5 conc. (µmol L^−1^)	Carbamoylase conc. (mg mL^−1^)	Reaction time (min)
1	0.3	0.2	30
2	3.5	0.2	5
3	0.3	2	5
4	3.5	2	30

Calibration measurements with the e-tongue with respect to the GTX5 and C1&2 were carried out in the mixture of solutions of enzyme and toxins prepared in buffer and bivalve extract free of toxins. Individual solutions of GTX5 and C1&2 were prepared in the 50 mmol L^−1^ phosphate buffer with pH 7.0 containing 5 mmol L^−1^ of NaCl in the concentration range from 0.05 to 1 µmol L^−1^. Mixed solutions of GTX5 and C1&2 were prepared in bivalve extracts free of PSTs diluted 4 times with ultrapure water in the concentration range from 0.025 to 0.5 µmol L^−1^ for each toxin. Contaminated bivalve extracts were diluted 4 times with ultrapure water. Prior to measurements, toxin solution and contaminated bivalve extracts were mixed with enzyme extract to obtain final enzyme concentration of 1 mg mL^−1^ and incubated for 5 min.

### FT-MIR spectroscopy

Enzyme solutions before and after incubation with substrate (GTX5) were analysed using Fourier transform mid-infrared (FT-MIR) spectroscopy. FT-MIR spectra were recorded using a MIR spectrometer (Bruker Alpha Platinum, Germany) equipped with an attenuated total reflectance accessory in a room with controlled temperature (22 °C) and humidity (40%). The enzyme solution was placed on the crystal, dried under cold air flow, and the spectra were acquired with a resolution of 4 cm^−1^ and 32 scans, in the mid-infrared region (4000–600 cm^−1^). For enzymatic reaction, 1 µmol L^−1^ of GTX5 was added to the enzyme solution with concentration of 1 mg mL^−1^ in 50 mol L^−1^ phosphate buffer with pH 7 and incubated for 30 min at 25 °C.

### Data processing

Capacitance values provided by the electrical measurements for frequencies from 100 Hz to 1 MHz (81 values in total) were used as analytical signal of the electronic tongue. Prior to all calculations, capacitance values were standardized.

The effects of the experimental factors (enzyme and toxin concentrations and incubation time) on the sensor response were evaluated using ANOVA simultaneous component analysis (ASCA). Two data sets were analysed: raw and subtracted data. Raw data were values of capacitance measured in solutions with different enzyme concentrations and in mixtures of enzyme and toxins with different concentrations of both. Subtracted data were measurements in the mixtures of enzyme and toxin from which capacitance values measured in enzyme solutions with corresponding enzyme concentration were subtracted. Subtraction was done to eliminate the effect of enzyme concentrations from electronic tongue response. The significance of the main effects of three factors was evaluated using a permutation test with 2000 permutations. The percentage of the variance explained by each factor or interaction was used as a quality-of-fit criterion [37]. Detailed description of the algorithms can be found elsewhere [37, 38].

Visualization of samples was done by principal component analysis (PCA).

Calibration models with respect to the concentration of individual toxins or sum of PSTs were calculated using partial least square regression (PLS) and validated using leave-one-out validation. Root mean square errors and adjusted *R*^2^ for calibration and validation data were used as figures of merit of the models.

Multivariate detection limit was determined using a procedure based on the IUPAC definition of low detection limit (LOD) adapted for the multivariate calibration as described in Ortiz MC et al. [39]. For the univariate case, minimum detectable net concentration, *x*_LOD_, is calculated using the following equation according to the ISO norm:

1 $${x}_{LOD}=\frac{{\delta }_{\alpha ,\beta }{S}_{blank}}{b}$$

where δ_α.β_ is the non-centrality parameter of the non-central *t* distribution, which is equal to 3 for α = β = 0.05, *S*_blank_ is standard deviation of the blank sample with analyte concentration 0, and *b* is a model parameter (sensitivity). The value of *S*_blank_ can be estimated by several independent measurements of blank solutions prepared in the same way as calibration samples. An alternative is to use the value of the standard deviation of the calibration line, *S*_y/x_, which is calculated using the following equation:

2 $${S}_{{}^{y}\!\left/ \!{}_{x}\right.}=\sqrt{\frac{\sum_{i}{\left({y}_{i}-\widehat{{y}_{i}}\right)}^{2}}{I-2}}$$

where *y*_i_ is the experimental signal in the samples used for calibration, $\widehat{{y}_{i}}$ the signal calculated by the model and *I* is the number of samples used in calibration.

Extension of the IUPAC definition to the multivariate calibration models is based on the assumption that the LOD is insensitive to any linear transformation applied to the signal. As was shown in Ortiz MC et al. [39], if data (*x*_i_, *y*_i_) are transformed into (*x*_i_, *y*_i_*) by means of *y*_i_* = *my*_i_ + *n*, the regression of Y* on the concentration *X* gives the same capability of detection, *x*_LOD_, as the regression of *Y* on the concentration *X*. This approach is applicable to linear multivariate methods such as PLS regression.

Thus, after PLS model is established, the analyte concentrations in the calibration samples are estimated by the PLS regression model and plotted against their nominal or measured concentrations. The resulting pseudo-univariate calibration graph, in which the vertical axis represents the estimated analyte concentration instead of either instrumental or latent variables, is used for calculating model parameter *b* using least square method and standard deviation *S*_y/x_ using formula (2). Both parameters are used for calculation of the detection limit using formula (1).

All algorithms were implemented in MATLAB® R2022b (Mathworks, Inc., Natick, MA, USA).

## Results and discussion

### FT-MIR characterization of enzymatic reaction

Changes in the carbamoylase conformation during enzymatic reaction were studied using FT-MIR spectroscopy. Infrared spectroscopy is an established technique for the analysis of secondary structure of proteins [40]. Amide I absorption band is considered to be the most useful for these studies as its main contribution comes from the C = O stretching vibration of the amide group (about 80%) with a minor contribution from the C-N stretching vibration. The exact position of this band is, thus, influenced by the strength of the hydrogen bonds involving C = O and C-N groups, thus reflecting protein secondary structure and making it particularly useful for the studies of protein aggregation, degradation, folding and unfolding [40].

**Fig. 1:**
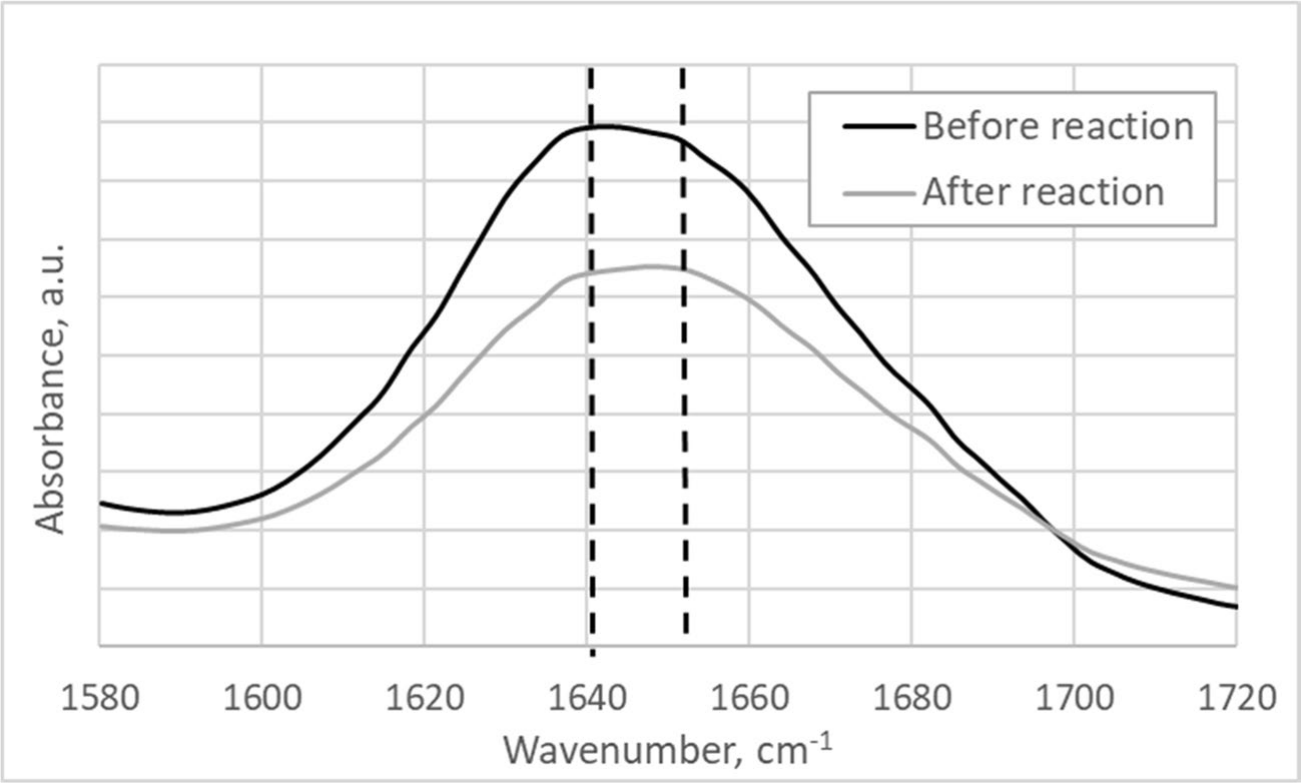
FTI-MIR spectra of carbamoylase before and after the reaction with the substrate (toxin GTX5)

FT-MIR spectra of carbamoylase before and after incubation with its substrate GTX5 in the spectral range corresponding to the amide I band are depicted in Fig. 1. Full FT-MIR spectra of carbamoylase before and after incubation with toxin are shown in Fig. 1S. The maximum of the amide I band of carbamoylase shifted from 1648 cm^−1^ before reaction to 1642 cm^−1^ after reaction. This suggests that the quantity of α-helixes or disordered structures (maximum at 1654 cm^−1^) decreased, while the quantity of β-sheets (maximum at 1633 cm^−1^) increased during enzymatic reaction, indicating conformational change and eventual enzyme degradation [40].

### EIS characterization of enzymatic reaction

Furthermore, conformational changes of the carbamoylase during hydrolysis of PSTs were evaluated using electrochemical impedance spectroscopy (EIS). EIS is a powerful technique for analysing surface phenomena and changes of bulk properties, both of which might be affected by the conformational changes of proteins [41]. The physical and chemical phenomena in an electrode-solution interface can be understood by modelling the capacitance (expressive of the charge separation in the interfacial region) in parallel with the resistance (resistance to charge movement).

The impedance of an electrode or modified electrode-solution interface can be directly measured by a sinusoidal small perturbation (10–100 mV of amplitude) to obtain a linear response, to simplify analysis of the spectra [42].

The complex impedance (${Z}^{*}$) can be written using the real (${Z}{'}$) and the imaginary (${Z}^{{'}{'}}$) parts of the impedance, according to Eq. (3):

3 $${Z}^{*}= {Z}{'}-i{Z}^{{'}{'}}$$

Nyquist plot (${Z}^{{'}{'}}=f({Z}{'})$) or a complex plane plot of impedance is often used for the data interpretation in the sensor applications [42]. As lower frequency phenomena are often more important in these studies, the main advantage of the Nyquist plot consists in turning features of the impedance plot at lower frequencies more evident than the ones at higher frequencies.

**Fig. 2:**
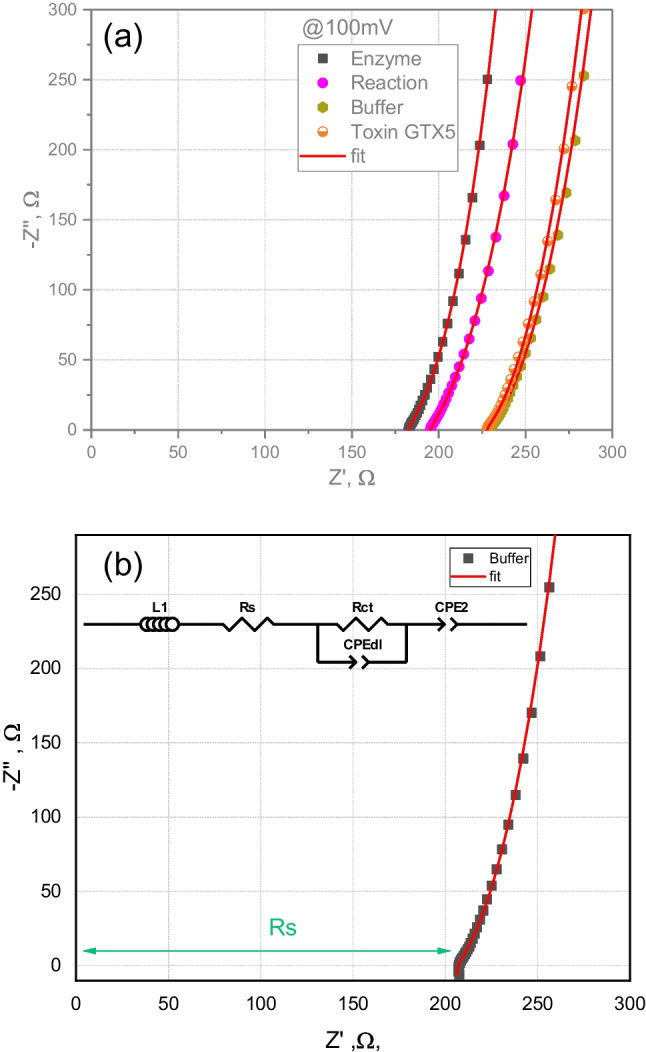
Complex plane impedance plot, showing the equivalent circuit fitting (red line) for **a** enzyme, reaction with substrate (GTX5), buffer and GTX5 solution in buffer, and **b** the equivalent circuit model used for fitting the impedance data for buffer, where *R*_s_ is the solution resistance, *R*_ct_ is the charger transfer resistance, CPE_dl_ and CPE_2_ are constant phase elements and L1 is an inductor

Complex plane impedance plots of the measurements in the enzyme solution, mixture of enzyme with substrate (GTX5), phosphate buffer and GTX5 solution (Fig. 2a) show similar behaviour and were modelled using the same equivalent circuit as the one shown in Fig. 2b.

The resistance *R*_s_ represents the sum of solution and cell resistances; nevertheless, as the contribution of the cell resistance is very low, the *R*_s_ is approximately equal to the solution resistance. The element L1 was added to the equivalent circuit model to account for the inductance present at high frequencies, which is related to the cable interference. Processes on the working electrode interfacial region are modelled by the charge transfer resistance, *R*_ct_, placed in parallel with the double-layer capacity, CPE_dl_. Constant phase element (CPE) is used instead of a pure capacitor as the complex plane plots do not show perfect semicircles that appear to be “depressed,” i.e. with the semicircle centre lying below the *x*-axis (Fig. 2a). This behaviour can be attributed to the non-uniformity, roughness and some porosity of the surface that in each microscopic area leads to different RC combinations [42]. What is observed macroscopically is the sum of all these contributions, which is often described using a constant phase element (CPE) for a non-ideal capacitor:

4 $${Z}_{CPE}=\frac{1}{{(i\omega )}^{\alpha }Q},$$

where $\omega$ is the angular frequency and *Q* and $\alpha$ are are the described parameters of CPE. Here, $\alpha$ is the CPE exponent, a dimensionless parameter that can take values from 0 to 1 [43]. When α = 1, CPE represents a pure capacitor, for α = 0.5, CPE is equivalent to the Warburg element, while for α = 0, the element is purely resistive. Furthermore, α values below 0.5 are related to highly depressed semicircles, which are not very common. The term *Q* does not have a clear physical meaning as capacitance; hence, after determining the CPE parameters and the resistance value (*R*) connected in parallel with the constant phase element, the corresponding capacitance can be estimated according to Brug’s formula (${\text{C}}={{\text{R}}}^{\frac{1-{\text{a}}}{{\text{a}}}}\cdot {{\text{Q}}}^{\frac{1}{{\text{a}}}}$) [44, 45].

The second constant phase element (CPE2) accounts for the low-frequency polarization, typically related to electrode polarization or the phenomena of Maxwell–Wagner–Sillars. The latter occurs in heterogeneous materials and is due to the charge accumulation at the interface of two materials with different charge carrier relaxation times [46]. Table 2 summarizes the values of resistance and capacitance of the analysed sample: enzyme solution, enzyme and GTX5 solution, phosphate buffer and GTX5 solution.

**Table 2:** Electrochemical parameters of an electrode in different solutions: enzyme, enzyme and toxin GTX5, phosphate buffer and toxin GTX5. *R*_s_ - solution resistance, *R*_ct_ – charge transfer resistance, *Q* and *α* - parameters of the constant phase elements, CPE_dl_ and CPE2, and *C*_dl_ - capacitance of electron transfer. Standard deviation is shown in the parentheses

Sample	*R*_s_, Ω	*R*_ct_, Ω	*R*_s_ + *R*_ct_, Ω	*Q*_CPEdl_, μFs^(α−1)^ cm^−2^	*α*_CPEdl_	*C*_dl_, µF cm^−2^	*Q*_CPE2_, μFs^(α −1)^ cm^−2^	*α*_CPE2_
Enzyme	180.7 (9.1)	51 (20)	232 (26)	115 (11)	0.6 (0.005)	1.9 (1.2)	1.20 (0.02)	1.000 (0.001)
Enzyme and GTX5	194.3 (9.1)	172 (20)	366 (26)	100 (11)	0.6 (0.005)	3.8 (1.2)	1.00 (0.02)	1.000 (0.001)
Buffer	220.6 (9.1)	254 (20)	475 (26)	142 (11)	0.5 (0.005)	7.0 (1.2)	0.90 (0.02)	1.000 (0.001)
GTX5	226.0 (9.1)	233 (20)	459 (26)	166 (11)	0.5 (0.005)	7.8 (1.2)	1.00 (0.02)	1.000 (0.001)

The constant phase element (CPE2) added to model the low-frequency impedance revealed *α* values between 0.96 and 1, a capacitive response close to a pure capacitor. This polarization phenomenon should be related to the ion-blocking nature of the gold electrode, resulting in the accumulation of charges at the sample–electrode interface.

**Fig. 3:**
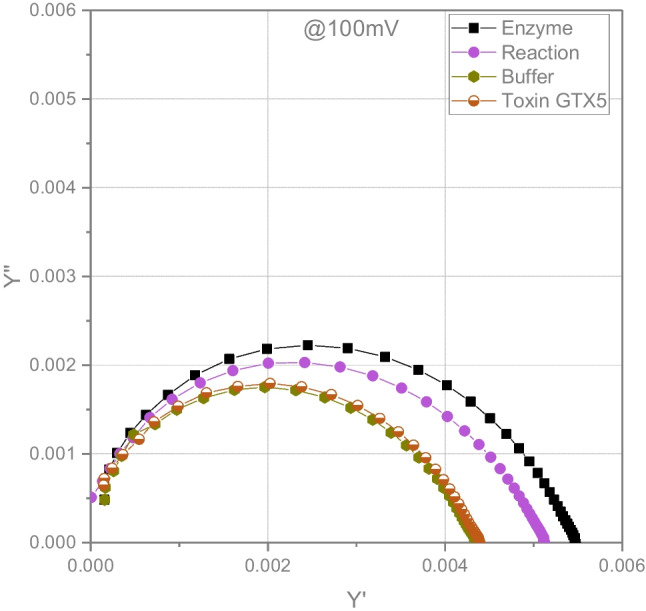
Complex plane admittance plot for different solutions: enzyme, enzymatic reaction (enzyme and GTX5), buffer and GTX5

The mathematic formalism of the complex admittance (${Y}^{*}= {Y}{'}+i{Y}^{{'}{'}}$) (Fig. 3), being the admittance the inverse of the impedance (${Y}^{*}=\frac{1}{{Z}^{*}}$), shows an asymmetric semicircle that could be attributed to the convolution of the charge transfer process (R_ct_ // CPE_dl_) and the low-frequency interfacial polarization. As expected, resistance values calculated using complex plane admittance and impedance were similar for all experimental conditions.

Results presented in Table 2 and Fig. 2a show that equivalent circuit parameters (*R*_s_, *R*_ct_, *Q*_CPEdl_, *α*_CPEdl_, *Q*_CPE2_, *α*_CPE2_ and *C*_dl_) for the phosphate buffer and toxin solutions do not differ significantly for an applied voltage of 100 mV. This can be ascribed to the fact that the toxin, which is not electroactive nor adsorbed on the electrode surface, is added to the buffer at low concentration and, thus, does not affect the properties of the system. *R*_ct_ electron transfer resistance and *C*_dl_ double-layer capacitance elements of the equivalent circuit in the phosphate buffer and toxin solution can be related to the gold corrosion process in the presence of chloride ions [47]. Slightly lower solution resistance *R*_s_ observed in the solution of enzyme and enzyme and toxins may be related to the presence of ions in the enzyme extracts from the purification step.

The largest differences in the equivalent circuit parameters were observed in the presence of enzyme and can be attributed to the enzyme adsorption on the electrode surface. The decrease of the double-layer capacitance *C*_dl_ compared to the pure metal electrode due to the electrode modifier with insulating properties has been described [24]. The increase of the *C*_dl_ in the enzyme solution in the presence of the substrate, GTX5, can be attributed to the alteration of the enzyme conformation during the reaction, which results in the decrease of the electrode surface insulation, corroborating results obtained using FT-MIR spectroscopy.

### Optimization of the electronic tongue working conditions

As the alteration of the enzyme conformation during enzymatic reaction can be detected by the EIS, enzymatic assay in combination with impedance spectroscopy can be employed for the sensing of the presence of toxins that are substrate of carbamoylase. The development of the impedimetric e-tongue has started with selection of the working electrode material and optimization of the assay conditions.

**Fig. 4:**
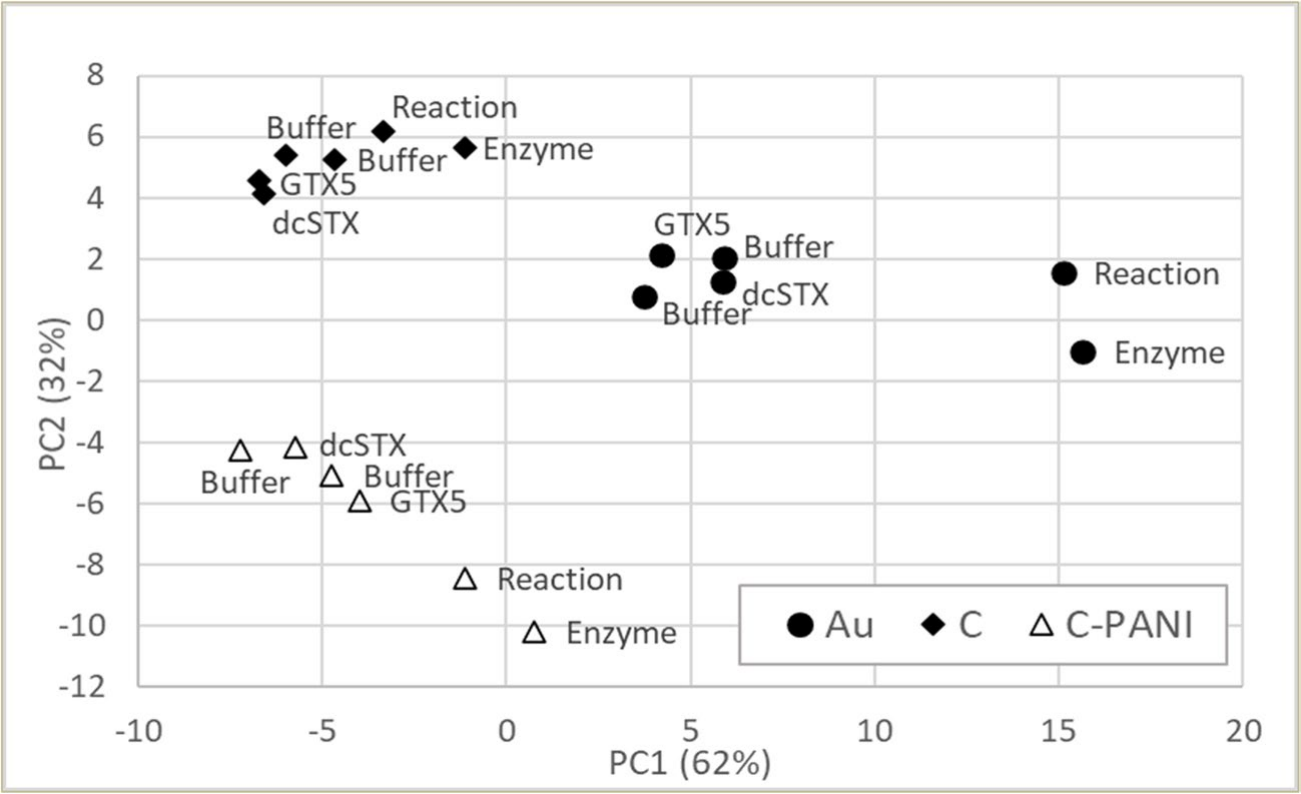
PCA score plot of measurements using sensors with three working electrodes: gold (Au), carbon (C) and carbon modified with polyaniline (C-PANI). Buffer, phosphate buffer; GTX5 and dcSTX, toxin solutions; enzyme, enzyme solutions; reaction, solution of enzyme and its substrate (GTX5)

The effect of the electrode material on the impedance spectra was evaluated using SPEs with working electrodes made of gold, carbon and carbon modified with polyaniline. Impedance spectra of the solutions of enzyme and toxins and a buffer were measured for the comparison. PCA score plot demonstrates that all three sensors allow to detect changes occurring during enzymatic reaction (Fig. 4). The difference between enzyme and enzyme and substrate solutions was similar for the gold, carbon and carbon modified with PANI electrodes, while the difference between solutions with and without enzyme was larger for the gold working electrode. Though all three electrode materials are suitable for the enzymatic assay, SPE with gold working electrode was used in further experiments.

Conditions of the enzymatic assay such as concentrations of enzyme and substrate (GTX5) and reaction time were optimized according to the experimental plan shown in Table 1. Results of the assessment of the statistical significance of the effects of the experimental factors on the e-tongue response using ASCA are presented in Table 3. According to the ASCA applied to the raw data, only the effect of enzyme concentration on the sensor response was statistically significant, accounting for 91.2% of the total variance (Table 3).

**Table 3:** Significance test of the effects of enzyme and toxin concentrations and incubation time on the electronic tongue response

Factor	Data set	*p*-value (2000 permutations)	Explained variance (%)
Enzyme conc	Raw data	0.021	91.2
Toxin conc		n.s	1.6
Incubation time		n.s	0.2
Enzyme conc	Subtracted data	n.s	19.7
Toxin conc		0.017	53.7
Incubation time		n.s	6.5

*n.s.* not statistically significant

The response parameter that is necessary to maximize for the analytical application is the difference between the e-tongue response in the solutions of enzyme and enzyme incubated with its substrate. Thus, ASCA was applied to the data set obtained by subtracting the response in the enzyme solution from the response in the mixture of enzyme with the same concentration and substrate. Only the effect of the toxin concentration was found to be statistically significant for the subtracted data set accounting for 53.7% of the total variance. This finding indicates the possibility of toxin quantification using impedimetric e-tongue besides simple detection of the PST presence (Table 3). As reaction time was not statistically significant, the shorter incubation time of 5 min was used in further assays.

**Fig. 5:**
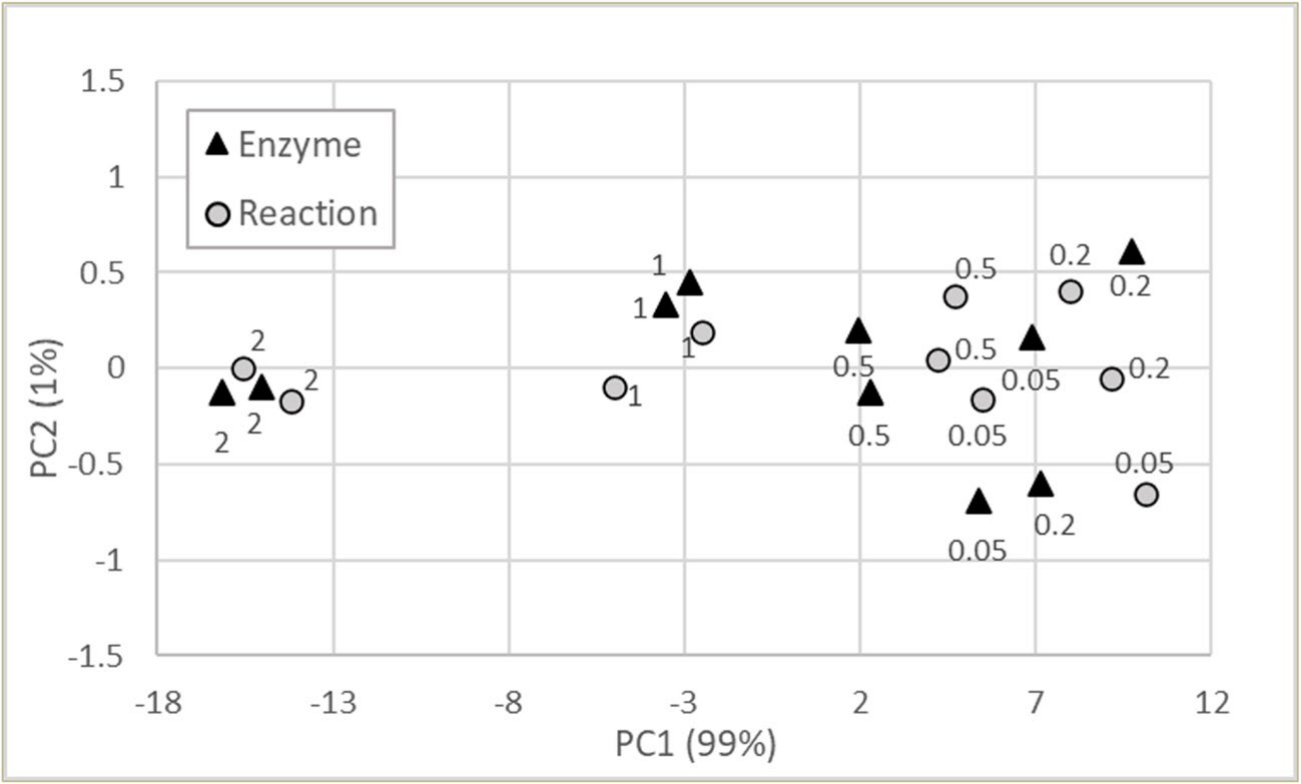
PCA score plot of measurements using gold electrode in the solutions of enzyme (enzyme) and mixture of enzyme and GTX5 toxin (reaction). GTX5 concentration was 0.3 µmol L^−1^; reaction time, 5 min; enzyme concentration in mg mL^−1^ is shown next to the symbols

As enzyme concentration was found to significantly affect e-tongue response (Table 3, raw data), further assays aiming to optimize enzyme concentration were carried out in the concentration range from 0.05 to 2 mg mL^−1^. PCA score plot demonstrates that enzyme concentration affects both the amplitude and reproducibility of the e-tongue response (Fig. 5). While differences between response in the enzyme and enzyme and substrate solutions were larger at lower concentrations of enzyme, reproducibility was poorer. As a compromise between reproducibility and the necessary amount of enzyme, the concentration of 1 mg mL^−1^ was selected for further experiments.

Other factors that may influence sensor response are temperature and variations of enzyme activity between different batches. Thus, temperature needs to be controlled during measurements preferably at around 25 °C, at which maximum carbamoylase activity was observed [20]. Activity variations can be accounted for by using enzyme from the same batch for both calibration and sample analysis.

### PSTs’ quantification using e-tongue

Response of the impedimetric e-tongue to the two N-sulfocarbamoyl PSTs, GTX5 and C1&2, both of which are carbamoylase substrates, was evaluated through calibration measurements in phosphate buffer spiked with either GTX5 or C1&2. Previously optimized assay conditions were used. Predicted vs. measured curves for two toxins are depicted in Fig. 2S. Root mean square errors, *R*^2^ and detection limits are presented in Table 4. The e-tongue could quantify both toxins in the concentration range from 0.1 to 1 µM with the detection limit of about 0.1 µM. As sum of the concentrations of N-sulfocarbamoyl toxins in the bivalve samples with toxicity close to regulatory limits is close to 0.5 µM, the obtained sensitivity is sufficient for the detection of these two PSTs in contaminated bivalve samples. Loading weights of the PLS calibration model shown in Fig. 3S indicate that response at low frequencies related to the interfacial processes has high importance for the model.

**Table 4:** Parameters of the predicted vs. measured curves for the calibration models, calculated using measurements in the phosphate buffer solutions and fourfold diluted uncontaminated mussel extract spiked with toxin standards. Calibration models were calculated using PLS regression and validated using leave-one-out validation

Sample	Toxin	RMSE, µmol L^−1^		*R*^2^		LOD, µmol L^−1^
		Calibration	Validation	Calibration	Validation	
Phosphate buffer	GTX5	0.036	0.088	0.992	0.950	0.11
	C1&2	0.018	0.10	0.998	0.939	0.064
Bivalve extract	GTX5 + C1&2	0.012	0.041	0.998	0.983	0.041

The validation of the impedimetric e-tongue for PST detection was carried out in the bivalve extracts. For this purpose, calibration measurements were made in the uncontaminated mussel extract spiked with toxin standards, after which the developed calibration model was used for PST quantification in contaminated mussel extracts.

Concentrations of PST toxins GTX5, C1&2, GTX6, dcSTX and dcGTX2&3 determined in the three composite mussel samples by the reference method (LC-FLD) [33] are shown in Table 5. Toxin concentrations in sample 1 were below the LOQs for all analysed toxins, most likely reflecting the absence or low density of toxic cells of *G. catenum* in the environment. Samples 2 and 3 showed relatively high concentrations of GTX5, C1&2, GTX6 dcSTX and dcGTX2&3, which are indicative of mussel exposure to a toxic algal bloom. STX and GTX2&3 concentrations were below quantification limits in all samples and were not included in the table. Toxin concentrations are in agreement with previous studies of the PST profiles in bivalves from Portugal [30, 48]. Total toxicity was estimated taking into account concentrations of all toxins (dcSTX, GTX5, C1&2, dcGTX2&3 and GTX6) currently measured in the reference laboratory [16]. The toxicity of both samples 2 and 3 exceeded the regulatory limit of 800 µg STX di-HCl equiv. kg^−1^. Toxins that are substrates of carbamoylase and can be detected by the impedimetric e-tongue, i.e. GTX5; GTX6 and C1&2 accounted for 73% and 70% of the total toxicity of samples 2 and 3, respectively.

**Table 5:** Concentrations (µmol L^−1^) of GTX5, GTX6, C1&2, dcSTX and dcGTX2&3 in mussel cleaned extracts determined by LC-FLD, total sample toxicity and contribution of GTX5, GTX6 and C1&2 to the total sample toxicity

Mussel samples	Toxin concentrations, µmol L^−1^						Toxicity, µg STX eq/kg
	dcGTX2&3	C1&2	dcSTX	GTX5	GTX6	GTX5 + GTX6 + C1&2	
**1**	nd	nd	nd	nd	nd	-	-
**2**	0.052	1.21	0.095	0.98	0.47	2.66	1161^*^ (1594^**^)
**3**	0.082	0.49	0.102	0.62	0.95	2.06	762^*^ (1089^**^)

^*^Sample toxicity related to GTX5, GTX6 and C1&2

^**^Total sample toxicity

As the e-tongue detects conformational changes induced by the carbamoylase binding with the substrate, any toxin that is a substrate of this enzyme can be detected. Furthermore, e-tongue cannot distinguish between different toxins that are carbamoylase substrates. In the case of toxin profile produced by *G. catenatum,* e-tongue would respond to the sum of N-sulfocarbamoyl toxins. Taking this into account, calibration measurements were made in the solutions spiked with two N-sulfocarbamoyl PSTs, GTX5 and C1&2, and the sum of their concentrations was used for calculating the calibration model. Thus, the uncontaminated bivalve extract (1) spiked with the GTX5 and C1&2 standards in the concentration range from 0.05 to 3 µmol L^−1^ was used for e-tongue calibration. The concentration range was selected to comprise the sum of the N-sulfocarbamoyl toxin concentrations in contaminated samples. As sensor response was found to become saturated at the concentrations above 1 µmol L^−1^ (data not shown), further calibration measurements were made in the extract diluted fourfold by ultrapure water and in the concentration range from 0.05 to 1 µmol L^−1^. Predicted vs. measured curves for the sum of GTX5 and C1&2 for the calibration solutions are shown in Fig. 4S. Root mean square errors, *R*^2^ and detection limits are presented in Table 4. Matrix compounds that may be present in bivalve extract did not affect the sensitivity of the impedimetric e-tongue; furthermore, lower detection limits of 0.04 µmol L^−1^ compared to the phosphate buffer were observed.

The obtained calibration model was applied to the detection of the sum of concentrations of PSTs that are carbamoylase substrates, which in the analysed extracts were N-sulfocarbamoyl toxins GTX5, GTX6 and C1&2. It should be noted that other toxins that are carbamoylase substrates, e.g. carbamate group STX and GTX2&3, could also be detected by the developed e-tongue. However, these two toxins were absent in the analysed contaminated mussel extracts as expected for the toxin profile originating from *G. catenatum* [48], and thus, they were not considered in this study. Two contaminated bivalve extracts were analysed by the e-tongue (extracts 2 and 3 in Table 3). Predicted sums of concentrations were 2.67 ± 0.17 and 2.11 ± 0.13 µmol L^−1^ for the extracts 2 and 3, respectively, and were not significantly different from the values obtained by the reference method according to the *t*-test (α = 0.05).

### Real-world applications and future research directions

The impedimetric e-tongue was demonstrated to be capable of detecting the presence of N-sulfocarbamoyl PSTs in the bivalve extracts. As N-sulfocarbamoyl PSTs account for approximately 70% of the total toxicity of bivalves, impedimetric e-tongue can be useful as a rapid screening method for bivalve toxicity assessment. Such features of the developed e-tongue as short analysis time, portability and inexpensive equipment make it attractive for use as a toxin screening tool including applications outside the laboratory. However, the necessity to use for each analysis a new portion of enzyme solution that needs to be stored refrigerated may hamper wider practical use of the developed e-tongue. Thus, future work shall focus on solving this issue. Among possible research directions are immobilization of the carbamoylase on the surface of the single-use sensor chip, which would allow eliminate use of enzyme solutions. Various approaches of enzyme immobilization can be considered including encapsulation, which has been reported to improve stability of the enzymes and their thermal stability in particular, providing more flexibility of modified sensor chip storage requirements [49].

Expedite and low-cost methodologies for the detection of PSTs have potential to be employed as screening tools in monitoring programs while also motivating producers to invest in self-control of toxins prior to export or sale to the market, thereby increasing trustworthiness of their activity. Due to sporadic occurrence of the HABs, in particular the ones associated with PSTs episodes, most of the samples analysed in the large-scale monitoring programs do not contain toxins above quantification limit. For example, 80% of the samples analysed in Portugal toxin survey program between 2003 and 2006 PST were not detected [50]. For this reason, a rapid screening tool capable to identify which samples may contain toxins and need to be re-analysed using reference methodologies would be very valuable for decreasing workload.

## Conclusions

An impedimetric e-tongue based on the gold sensor and enzymatic assay was proposed for the detection of major N-sulfocarbamoyl PSTs, GTX5, GTX6 and C1&2. The study of the carbamoylase reaction with PSTs by FT-MIR spectroscopy and electrochemical impedance spectroscopy revealed conformational changes occurring during enzymatic reaction that enabled the PSTs’ quantification. After optimization of the measurement conditions, the e-tongue could quantify N-sulfocarbamoyl PSTs with the detection limit of 0.1 µmol L^−1^, which reaches the detection of these toxins at the concentration levels observed in the extracts of bivalves with PST toxicity close to the regulatory limit. The sum of the N-sulfocarbamoyl PST concentrations detected in naturally contaminated mussel extracts by the impedimetric e-tongue was close to values as obtained by the reference method, LC-FLD. Impedimetric e-tongue can be a useful tool for rapid screening of PST toxicity in bivalve extracts.

## Supplementary Information

Below is the link to the electronic supplementary material.

Supplementary file1 (DOCX 96 KB)
