# Supplementary material for: Carbamoylase-based impedimetric electronic tongue for rapid detection of paralytic shellfish toxins
Source: Anal Bioanal Chem. 2024 Feb 15;416(8):1983–95. doi: 10.1007/s00216-024-05199-8 (PMC11461580; doi:10.1007/s00216-024-05199-8)

**Supplementary material.**

**Figure 1S.** Full FT-MIR spectra of carbamoylase before and after the reaction with the substrate (toxin GTX5).


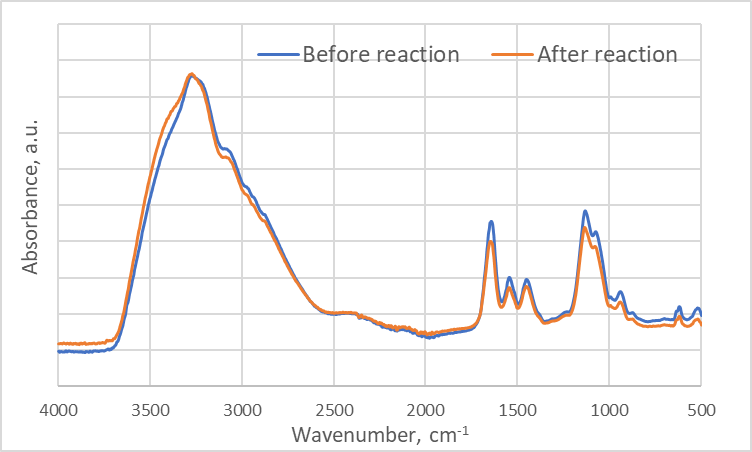


**Figure 2S.** Predicted vs. measured concentration plots for toxins C1&2 and GTX5. Measurements were made in the 1mM phosphate buffer solutions with pH 7 and with enzyme concentration 1 mg mL^-1^ and reaction time 5 minutes.

| 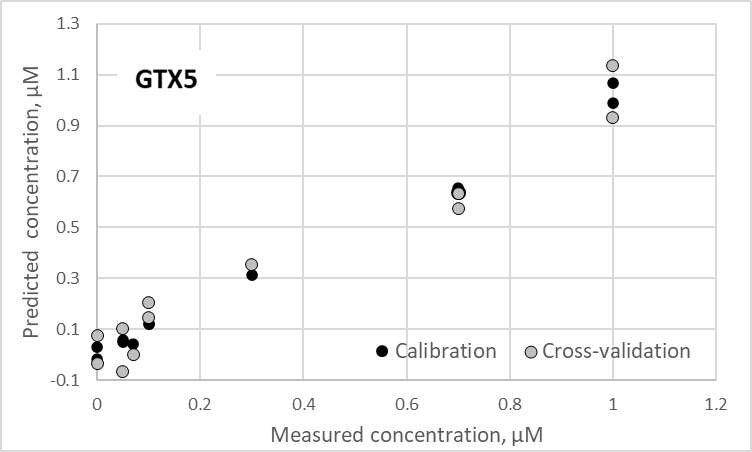 | 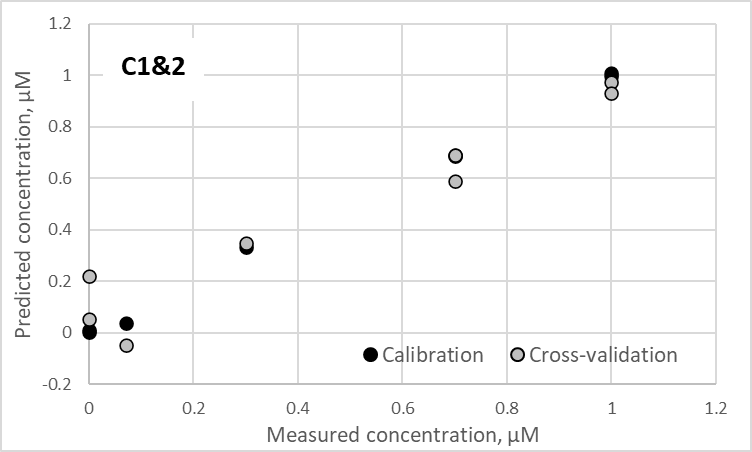 |
| --- | --- |

**Figure 3S.** Loading weights for the PC 1 of the PLS calibration model calculated using measurements in the phosphate buffer solutions extract spiked with GTX5 standards.

**
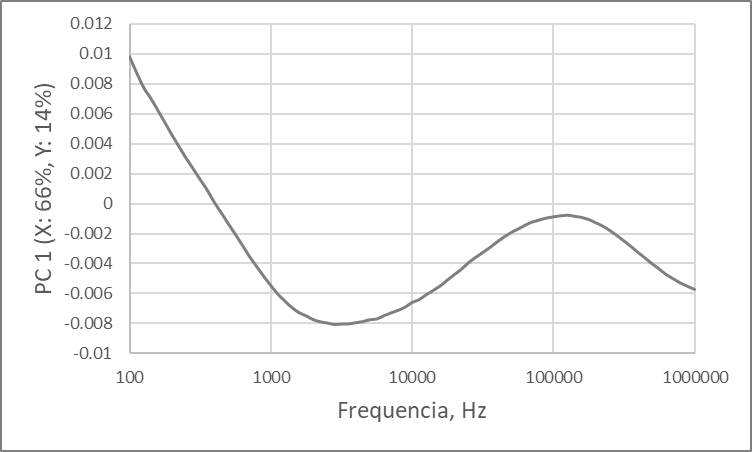
**

**Figure 4S** Predicted vs. measured concentration plots for the sum of toxins C1&2 and GTX5. Measurements were made in the uncontaminated mussel extract diluted 4-fold with ultrapure water, with enzyme concentration 1 mg mL^-1^ and reaction time 5 minutes.


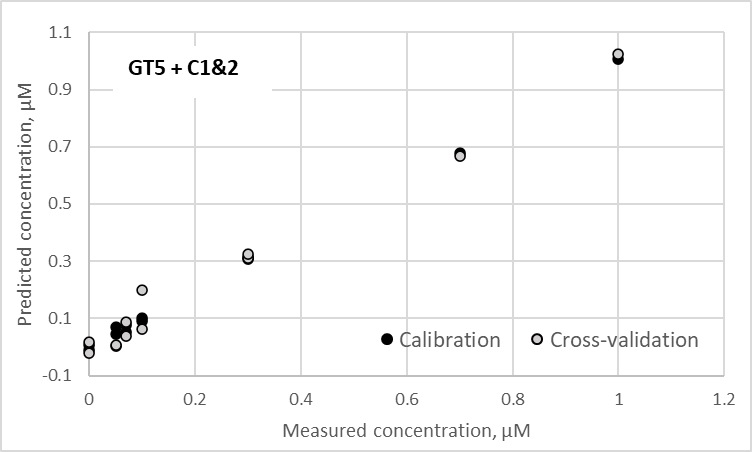

Supplement: Supplementary file 1 — Supplementary file1 (DOCX 96 KB) [file 216_2024_5199_MOESM1_ESM.docx]
